# Editorial: Genetic and molecular determinants in bone health and diseases

**DOI:** 10.3389/fendo.2024.1347765

**Published:** 2024-01-17

**Authors:** Michela Rossi, Jonathan W. Lowery, Andrea Del Fattore

**Affiliations:** ^1^ Bone Physiopathology Research Unit, Translational Pediatrics and Clinical Genetics Research Division, Bambino Gesù Children’s Hospital, IRCCS, Rome, Italy; ^2^ Division of Academic Affairs, Marian University, Indianapolis, IN, United States; ^3^ Department of Physiology & Pharmacology, College of Osteopathic Medicine, Marian University, Indianapolis, IN, United States; ^4^ Bone & Muscle Research Group, Marian University, Indianapolis, IN, United States; ^5^ Indiana Biosciences Research Institute, Indianapolis, IN, United States; ^6^ Indiana Center for Musculoskeletal Health, Indiana University School of Medicine, Indianapolis, IN, United States

**Keywords:** bone, osteoclast, osteoblast, bone disease, gene

Alterations of bone remodeling impact skeletal integrity lead to excessive or impaired bone resorption as well as reduced or disorganized bone formation (1, 2). This Research Topic focuses on the identification of genetic and molecular determinants involved in both bone health and diseases. In this editorial, we highlight studies on rare diseases presented in the Research Topic with the aim of better understanding their etiopathogenesis and opening the way for the identification of new therapeutic approaches.

Xiang and Zhong summarized the recent studies regarding the molecular and cellular mechanisms leading to the progressive osteolysis and angiomatous proliferation in Gorham-Stout disease (GSD), which is a very rare disease that is also known as Vanishing Bone Disease. GSD is characterized by severe osteolytic bone destruction but lacks specific diagnostic markers and therapy (3). The information presented by Xiang and Zhong provides an important update on the condition and presents ideas for new therapeutic approaches for this rare disease.

Cinque et al. published an elegant study on hypophosphatasia (HPP), a rare genetic disease affecting bone and teeth mineralization with multisystemic manifestations involving the nervous system, musculoskeletal apparatus, and kidneys, due to *ALPL* mutations. The authors reported the genetic analysis performed on 33 patients, identifying eight novel variants of *ALPL* gene. These results associated with the detailed clinical description increase the knowledge of this rare condition.

Osteogenesis imperfecta (OI), also known as brittle bone disease, is a clinically and genetically heterogeneous disorder of connective tissue and is identified by bone dysplasia and fragility (4). In this Research Topic, Paduano et al. reported the results obtained by next-generation sequencing (NGS) analysis of 10 patients, comprising 7 male and 3 female patients from 7 families, all from the Puglia Region in South Italy. The authors identified novel rare pathogenic variants in type I collagen-encoding genes (*COL1A1* and *COL1A2*).

In another study regarding OI, Lim et al. described the effects of a missense variant of *MBTPS2 –* which encodes the site-2 protease, a Golgi transmembrane protein that activates membrane-tethered transcription factors – in aborted male fetus with micromelia particularly of the lower limbs, a narrow thorax, and defective ossification of calvarium. The authors performed *in vitro* studies on mutated *MBTPS2* primary fibroblasts and found perturbations in fatty acid metabolism and collagen production.

Sundqvist et al. report a case study on rare, chronic non-bacterial osteomyelitis (CNO). They described a female patient with CNO with systemic inflammation, advanced malnutrition and complete deficiency of myeloperoxidase (MPO). The authors reported that, although the patient did not find beneficial effects after treatment with nonsteroidal anti-inflammatory drugs, corticosteroids, bisphosphonates or IL1-receptor antagonists (anakinra), the administration of TNFα blockade (adalimumab) resulted in instant resolution of the inflammatory symptoms suggesting that the disease was TNFα-driven.

Bone tissue is tightly connected with other organs to regulate whole physiology (5). In this Research Topic the interplay bone-liver has been reported. Huang et al. investigated whether serum liver enzymes are causally associated with bone and joint-related diseases using Mendelian randomization (MR) designs. Indeed, the positive causality between ALP and the risk of osteoporosis and rheumatoid arthritis was indicated. Moreover, the authors reported that higher levels of alanine transaminase (ALT) were associated with the risk of hip and knee osteoarthritis while no causal relationship between GGT and bone and joint-related diseases was revealed.

Moreover, two further papers reported new advances in bone remodelling, using animal models. Verlinden et al. investigated how neuropilin 2 (NRP2) in osteoblasts regulates trabecular bone mass in male mice. NRP2 is a non-tyrosine kinase transmembrane glycoprotein receptor. The authors generated two different genetic models lacking Nrp2 expression in osteoblasts or osteoclasts to identify its role in the bone remodelling activity. Although loss of *Nrp2* in the osteoclast lineage did not result in a bone phenotype, loss of *Nrp2* in osteoblast precursors and mature osteoblasts leads to reduced cortical cross-sectional tissue area and lower trabecular bone content in male mice.

Li et al. performed the evaluation of bone turnover markers and DEXA (Dual-Energy X-Ray Absorptiometry) analysis in cynomolgus monkeys at different ages to establish an animal model for age-related osteoporosis in non-human primates. The authors find that, in cynomolgus monkeys, peak BMD occurs at age 10 years of age then plateaus until old age, with a trend of bone turnover markers similar to that of humans.

In conclusion, the papers published in this Research Topic underline how investigating bone diseases and animal models represent a way to find new determinants of bone physiology and also allow the identification of new therapeutic approaches.

## Author contributions

MR: Writing – original draft, Writing – review & editing. JL: Writing – original draft. AD: Writing – original draft, Writing – review & editing.
